# Personalized colorectal cancer risk assessment through explainable AI and Gut microbiome profiling

**DOI:** 10.1080/19490976.2025.2543124

**Published:** 2025-08-04

**Authors:** Pierfrancesco Novielli, Simone Baldi, Donato Romano, Michele Magarelli, Domenico Diacono, Pierpaolo Di Bitonto, Giulia Nannini, Leandro Di Gloria, Roberto Bellotti, Amedeo Amedei, Sabina Tangaro

**Affiliations:** a Department of Soil, Plant and Food Sciences, University of Bari Aldo Moro, Bari, Italy; b National Institute for Nuclear Physics, Bari Division, Bari, Italy; c Department of Experimental and Clinical Medicine, University of Florence, Florence, Italy; d Department of Biomedical, Experimental and Clinical Sciences “Mario Serio”, University of Florence, Florence, Italy; e Interuniversity Department of Physics “M. Merlin”,University of Bari Aldo Moro, Bari, Italy; f Network of Immunity in Infection, Malignancy and Autoimmunity (NIIMA), Universal Scientific Education and Research Network (USERN), Florence, Italy; g Laboratorio Congiunto MIA-LAB (Microbiome-Immunity Axis Research for a Circular Health), University of Florence, Florence, Italy

**Keywords:** Explainable AI, microbiome, colorectal cancer, biomarker, SHAP interaction analysis, risk stratification

## Abstract

The clinical adenoma – carcinoma progression represents a well-established framework for understanding colorectal cancer (CRC) development, although the molecular mechanisms underlying this transition remain only partially understood. Increasing evidence suggests the gut microbiome (GM) as a key modulator of colorectal carcinogenesis, positioning microbial profiling as a promising avenue for noninvasive risk stratification and early detection. In this study, Machine Learning (ML) classifiers integrated with eXplainable Artificial Intelligence (XAI) techniques were employed to identify microbiome-derived biomarkers predictive of CRC and adenomatous lesions. The models were trained on 16S rRNA sequencing data from 453 patients and evaluated through cross-validation, achieving AU-ROC and AU-PRC scores of 0.71 and 0.67, respectively. External validation on an independent Italian cohort (n=43) yielded AU-ROC and AU-PRC scores of 0.70 and 0.89, respectively. XAI-based interpretation revealed consistent microbial signatures across datasets. In detail, taxa belonging to the *Fusobacterium* and *Peptostreptococcus* genera were associated with increased CRC risk, whereas the *Eubacterium eligens* group was identified as a robust negative predictor. Beyond classification, patient-level explanations enabled by XAI facilitated the identification of adenoma subgroups exhibiting microbiome profiles converging toward those of CRC, suggesting the presence of transitional microbial states. Moreover, SHAP-based interaction networks uncovered microbial hubs and inter-species dependencies characterizing high-risk configurations, providing insights into the ecological dynamics of colorectal tumorigenesis. These findings demonstrate the added XAI value in elucidating microbiome interactions, enhancing model interpretability, and supporting biologically informed hypotheses. This integrative, explainable framework highlights the potential of AI-driven microbiome analysis in precision oncology and advances the development of interpretable, noninvasive tools for CRC risk prediction and management.

## 1. Introduction

Colorectal cancer (CRC) is a significant global health challenge, ranking among the most prevalent malignancies in adults and a leading cause of cancer-related mortality worldwide.^1^ The CRC pathogenesis involves a well-defined sequence of events, beginning with normal colonic epithelium progressing to adenoma and lastly to invasive carcinoma.^2–5^ This adenoma-carcinoma provides a critical framework for understanding CRC onset and development^6^; however, despite substantial progress, the intricate molecular mechanisms underlying this progression remain incompletely understood.^7^ Currently, colonoscopy is the gold standard for CRC diagnosis and screening. While highly effective, its invasive nature and associated patient discomfort limit widespread compliance. This limitation underscores the pressing need for noninvasive approaches to enable early detection and risk stratification of CRC. Emerging research has highlighted the critical role of the gut microbiome (GM) – the diverse and dynamic community of microorganisms residing in the host’s gastrointestinal tract – in the modulation of colorectal carcinogenesis.^8–13^

Recent studies have further emphasized the influence of the GM on host health and disease trajectories across various clinical and biological contexts. Bai et al. highlighted how hydrolyzed protein formulas modulate intestinal development and reshape microbial communities in low-birth-weight piglets, pointing to diet as a key modulator of host – microbe interactions.^14^ Fan et al. conducted a comparative GM analysis in peritoneal dialysis patients, revealing microbial differences between incident and prevalent cases and their association with peritoneal membrane function.^15^ Moreover, Chen et al. integrated microbiome and serum metabolome profiling to identify distinctive microbial and metabolic signatures in patients with colorectal polyps, providing novel insights into early, noninvasive CRC risk detection.^16^ Lastly, Huang et al. employed multi-omics approaches to show how host genetic background influences colonic microbiota composition across pig breeds.^17^

Building on these insights, multiple studies have sought to unravel the complex interactions between intestinal bacteria and CRC progression. Specifically, *Fusobacterium nucleatum* has been implicated in CRC due to its ability to promote inflammation, modulate immune responses, and promoting tumorigenesis by binding to E-cadherin and activating the β-catenin signaling pathway.^18^ Similarly, polyketide synthase-positive *Escherichia coli* strains produce colibactin, a genotoxic protein that causes deleterious effects on DNA within host colonocytes.^19^

In this scenario, a burgeoning body of research is dedicated to leveraging Machine Learning (ML) techniques to explore the complex relationships among the GM and host health, with the goal of identifying microbial biomarkers indicative of CRC progression.^20–23^ In detail, the integration of eXplainable Artificial Intelligence (XAI) methodologies has significantly enhanced the interpretation of complex microbiome datasets, facilitating the extraction of biologically meaningful insights and the identification of critical disease-associated microbial signatures. Furthermore, the integration of XAI analysis with SHAP (SHapley Additive exPlanations) values, providing an in-depth understanding of how individual features influence the predictions of a ML model, not only simplify the identification of novel biomarkers linked to disease progression but also pave the way for developing novel embeddings of microbiome data.^24–27^

Beyond microbiome-centric studies, recent research highlights the growing relevance of interpretable AI and computational frameworks across diverse clinical domains. Li et al. developed a deep learning framework for automatic ulcerative colitis severity assessment from endoscopic images, integrating Grad-CAM (Gradient-weighted Class Activation Mapping) to provide visual interpretability of model predictions and enhance clinical trust.^28^ Hu et al. proposed an integrated network pharmacology framework that combines microbiome-derived metabolism and hepatic biotransformation to elucidate the therapeutic mechanisms of bioactive compounds in neurological disorders, using Astragaloside IV as a case study in intracerebral hemorrhage.^29^ Although applied to distinct biological systems and data types, these studies share the goal of using interpretable or systems-level computational tools to uncover complex biological patterns and support precision diagnostics. This broader context underscores the translational potential of explainable AI in various biomedical applications, including the GM evaluation in CRC.

While several high-impact studies have applied metagenomic approaches to identify microbiome-based biomarkers for CRC detection and staging – notably Yachida et al.,^30^ Thomas et al.,^31^ and Wirbel et al.^32^—these works primarily employed shotgun sequencing to characterize microbial shifts across CRC stages or populations, achieving high classification accuracy but offering limited interpretability at the individual level. The present study introduces a complementary 16S rRNA-based framework, uniquely integrating explainable machine learning (SHAP), unsupervised clustering, and interaction network analysis to stratify adenoma and CRC patients. This approach enables both robust classification and mechanistic insight into patient-specific microbial risk profiles, with potential applications in noninvasive precision diagnostics.

Hence, this study aims to couple ML techniques with XAI methodologies to further unravel the complex interactions between GM and CRC onset and progression enhancing diagnostic precision and prognostic insights.

## 2. Materials and methods

This study employed three publicly available datasets from previous research studies, encompassing a total of 453 patients diagnosed with adenoma (adenoma) or CRC, sourced from diverse geographic regions, including Canada (CA), France (FRA), and the United States of America (USA).^33–35^ Each dataset included V4 16S rRNA gene sequencing data of fecal microbiota, enriched with metadata attributes such as gender, age and body mass index (BMI), as summarized in Table 1. These metadata allowed for a comprehensive investigation of the associations between GM composition and clinical parameters across adenoma and CRC patient cohorts.

**Table 1.** Demographic characteristics of the study participants. The Fisher’s exact test was performed for gender and country; the Mann-Whitney U rank test for age and BMI.

	CRC (189)	Adenoma (264)	*p*-value
Gender	77F/114M	109F/155M	0.85
Country	2 CA/41 FRA/146 USA	25 CA/37 FRA/202 USA	<0.01
Age	62.58 ± 12.59	62.47 ± 10.65	0.77
BMI	28.23 ± 6.15	26.54 ± 4.63	<0.01

The original studies from which the datasets were obtained defined consistent inclusion and exclusion criteria to ensure sample quality and comparability. Common exclusion criteria across the studies included inflammatory bowel disease, prior surgery or treatment for CRC or adenomas, and hereditary cancer syndromes such as familial adenomatous polyposis (FAP) or Lynch syndrome (HNPCC).^33–35^ Only patients aged 18 years or older, with histologically confirmed diagnoses and available metadata (age, sex, BMI), were included in our analysis.

To validate the predictive model developed in this study an independent test set, distinct from the training data, was employed.^36^ This test dataset, detailed in Table 2, consisted of 43 Italian (ITA) patients, including 34 diagnosed with CRC and 9 with adenoma. The samples underwent the same preprocessing and analysis steps as the training data, ensuring consistency across the analysis. This independent validation was crucial for evaluating the model’s generalization ability in a geographically distinct population, reducing the risk of overfitting and confirming the robustness of the predictive framework.

**Table 2.** Demographic characteristics of the independent test set. The Fisher’s exact test was performed for gender and country; the Mann-Whitney U rank test for age and BMI.

	CRC (34)	Adenoma (9)	*p*-value
Gender	5F/29M	4F/5M	0.07
Country	34 ITA	9 ITA	0
Age	71.38 ± 12.28	62.67 ± 12.63	0.04
BMI	26.3 ± 4.33	25.57 ± 2.35	0.55

**Figure 1.**
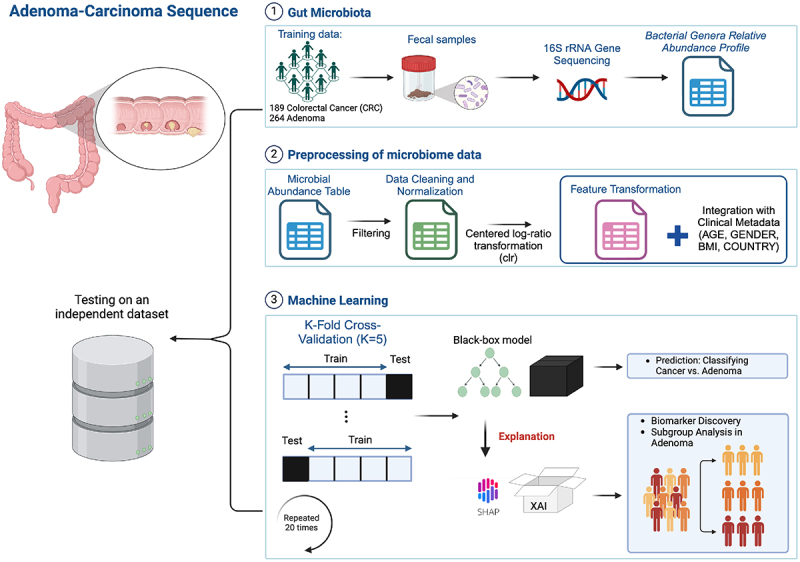
Experimental workflow of the study. Publicly available 16S rRNA gene sequencing data was used to analyze the adenoma-carcinoma sequence in GM profiles. The workflow starts with preprocessing the GM abundance data and integrating it with clinical metadata (age, BMI, gender, country). Three tree-based machine learning models (XGBoost, Random Forest, and CatBoost) were trained to classify CRC and adenoma cases, followed by SHAP-based interpretation for feature importance and risk subgroup identification in adenoma subjects. The models were further validated on an independent dataset to assess generalization performance.

The experimental workflow followed for the analysis is depicted in Figure 1.

The initial step of the analysis involved preprocessing the GM abundance data derived from 16S rRNA gene sequencing, with a focus on cleaning and transforming the data to prepare it for use in ML classification models. To enrich the predictive analysis, demographic data such as age, BMI, and country of origin were integrated with the GM data, creating a comprehensive dataset. Three tree-based classification models, namely XGBoost, Random Forest, and CatBoost, were subsequently evaluated to identify the most effective classifier. Model interpretability was further enhanced through the calculation of SHAP values, providing insights into the model’s predictions and identifying potential biomarkers. Specifically, SHAP analysis facilitated the making of a new embedding space for clustering adenoma patients based on their GM profiles. Model testing on an independent dataset validated its generalization capability, confirming robustness and applicability across different populations.

### 2.1. Preprocessing

Preprocessing of sequencing data stands as a critical step in the analysis pipeline, essential for ensuring the accuracy and reliability of downstream analyses.^37,38^

The first preprocessing step involved taxonomic unit filtration, where non-informative features or biologically irrelevant taxa, including potential contaminants, were excluded.^39^ Thresholds can be set on criteria such as abundance, prevalence, variance, or correlation; in this case, a prevalence threshold was applied, retaining only taxa detected in at least 10% of the samples.

Subsequently, normalization was applied to correct for variability in sequencing depth and data sparsity. Given the compositional nature of GM datasets, Aitchison’s methodology for compositional data analysis was applied,^40,41^ transforming feature counts into log-ratios within each sample using an additive, centered log-ratio transformation.

### 2.2. ML classifiers

The selected tree-based machine learning models, known for their proven efficiency in analyzing tabular data,^42,43^ were XGBoost, Random Forest, and CatBoost. All three tree-ensemble algorithms include built-in mechanisms that automatically attenuate uninformative variables: splits occur only on predictors that lower impurity, while column subsampling (Random Forest), learning-rate shrinkage with $\ell_{1}/\ell_{2}$ penalties (XGBoost), and ordered boosting with Bayesian smoothing (CatBoost) further regularize the models.^44–46^ These safeguards limit overfitting, and benchmarks confirm that tree ensembles excel on medium-sized tabular data with minimal feature engineering.^43,47^ The performance of these algorithms was evaluated using three metrics: accuracy, Area Under the Receiver Operating Characteristic Curve (AU-ROC), and Area Under the Precision-Recall Curve (AU-PRC). Accuracy was used to assess the proportion of correctly classified instances among the total instances. AU-ROC was employed to evaluate the effectiveness of binary classification models by quantifying the area under the ROC curve. In this study, the ROC curve illustrates the trade-off between the true positive rate and the false positive rate across different classification thresholds, where CRC instances were considered positive and adenoma instances were considered negative. Additionally, the PRC its corresponding AUC were used to evaluate model performance. The PRC illustrates the relationship between precision and recall at various decision thresholds. Precision measures the proportion of instances predicted as CRC that are actually CRC, while recall measures the proportion of actual CRC instances correctly identified by the model. The Area Under the PRC Curve provides an aggregated measure of the model’s performance in terms of both precision and recall.

### 2.3. XAI analysis: SHAP values

The XAI framework encompasses various techniques aimed at enhancing informativeness, uncertainty estimation, generalization, and transparency. The SHAP local explanation algorithm was adopted to unveil the significance of features in classifying adenoma and CRC samples. Acting as a local, model-agnostic post-hoc explainer, the SHAP algorithm draws inspiration from Shapley values rooted in cooperative game theory.^48,49^ SHAP constructs interpretable linear models for individual samples, clarifying the contribution of each feature to the sample’s prediction. The computation of SHAP values involves evaluating the difference in model’s output predictions with and without specific features. As a result, the method requires retraining the model on all subsets F of the complete set S of features (F⊆S). The SHAP value for the $j^{\mathrm{th}}$ feature of a given instance x is calculated by aggregating its contributions across all possible subsets:

(1) $$\Phi_{j}(x)=\sum_{F\subseteq S-\{j\}}\frac{|F|!(|S|-|F|-1)!}{|S|!}[f_{x}(F\cup j)-f_{x}(F)]$$

where |F|! represents the permutations of features in the subset F, (|S|−|F|−1)! the permutations of features in the subset S−(F∪{j}), and |S|! is the total number of feature permutations.

The use of SHAP values enabled the construction of a feature relevance ranking to identify the most critical bacterial taxonomic units in the adenoma-CRC sequence. Additionally, SHAP values were used to visualize the analyzed patients. To handle the numerous available variables, a data reduction algorithm, t-SNE (t-distributed Stochastic Neighbor Embedding)^50,51^ was applied, serving as an effective tool for visualizing high-dimensional datasets. Following dimensionality reduction with t-SNE, three different clustering methods were applied to group the adenoma subjects: K-means clustering,^52^ Agglomerative Clustering, and Birch (Balanced Iterative Reducing and Clustering using Hierarchies) Clustering. K-means clustering partitions the data into a predefined number of clusters by minimizing within-cluster variance. In contrast, Agglomerative Clustering,^53^ a hierarchical method, iteratively merges the closest data points into clusters based on a distance metric. Birch Clustering^54^ organizes data into compact subclusters, facilitating efficient large-scale clustering with low memory requirements. To conclude, the silhouette score was utilized to determine the optimal number of clusters and select the most suitable clustering method.^55^ This metric evaluates the quality of clustering by balancing cluster cohesion and separation, ensuring robust grouping for the analysis of adenoma subjects.

### 2.4. SHAP interaction analysis

To extend the analysis, SHAP interaction values were calculated to investigate the pairwise interactions between features, using the TreeExplainer and shap_interaction_values functions from the SHAP package. The SHAP interaction value between two features, *i* and *j*, quantifies how their combined presence influences the model’s output prediction, compared to the contributions of each feature independently. High SHAP interaction values indicate strong dependencies or interactions between features.^56^

The SHAP interaction analysis was performed during the repeated 5-fold cross-validation process using the best-performing ML model identified in the evaluation. For each fold, interaction values were averaged across samples to generate a fold-specific interaction matrix. These matrices were then aggregated and averaged over all folds to create a final interaction matrix, representing the absolute mean SHAP interaction values across the entire cross-validation process.

The distribution of SHAP interaction values was visualized through violin plots to identify the most influential feature interactions. To retain only the most robust interactions, a percentile-based threshold was applied. This step ensured a focused analysis of meaningful interactions while filtering out noise.

Next, a weighted interaction network was constructed using the selected features and their SHAP interaction values. In this network, nodes represent features, and edge weights correspond to their SHAP interaction values. Network analysis was conducted to extract metrics such as degree, betweenness centrality, and interaction counts for each feature, providing insights into the microbiome’s interaction dynamics and its influence on the adenoma-CRC sequence.

The entire workflow was implemented in Python.

## 3. Results

The aim of this study was to build a ML classifier that predict adenoma/CRC status from GM profiles. After applying a 10% prevalence filter, the microbiome feature set shrank from 462 to 164 genera, which were combined with four clinical covariates. Three tree-ensemble algorithms – Random Forest, XGBoost and CatBoost – were compared within a unified pipeline, and SHAP values were computed to interpret model decisions.

The performance of the three models in terms of accuracy, AU-ROC, and AU-PRC are summarized in Table 3. The reported values are based on a Stratified K-Fold (K = 5), repeated five times to ensure robustness. The results are represented as mean ± standard deviation across 20 repetitions. Of note, the results documented comparable performance among the models, with no single algorithm demonstrating statistically superior outcomes. For subsequent analyses, CatBoost was selected due to its consistency, although Random Forest and XGBoost yielded similarly robust results.

**Table 3.** Comparison between evaluation metrics of XGBoost (XGB), Random Forest (RF), and catboost classifiers. The mean values accompanied by the standard deviation are shown.

ML Classifier	Accuracy	AU-ROC	AU-PRC
XGBoost	0.67 (0.01)	0.69 (0.02)	0.64 (0.02)
Random Forest	0.68 (0.01)	0.70 (0.01)	0.66 (0.01)
CatBoost	0.69 (0.01)	0.71 (0.01)	0.67 (0.01)

Specifically, the CatBoost classification achieved an accuracy of 0.69 ± 0.01, an AU-ROC of 0.71 ± 0.01, and an AU-PRC of 0.67 ± 0.01.

**Figure 2.**
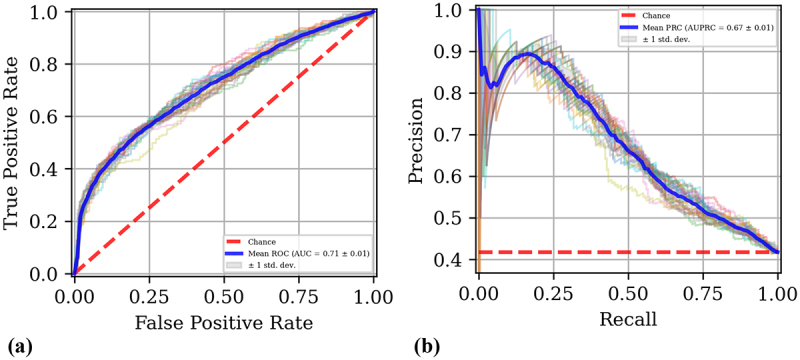
Receiver operating characteristic (ROC) and precision-recall (PR) curves for the CatBoost model. (a) ROC curve illustrating the trade-off between true positive rate and false positive rate. (b) PR curve showing the relationship between precision and recall.

Figure 2(a,b) display the ROC curve and the PR curve of the CatBoost model, respectively.

To validate the generalizability of the CatBoost model, it was tested on the ITA independent dataset, which consisted of 34 CRC and 9 adenoma diagnosed patients. The evaluation utilized all models trained during the cross-validation phase (100 models in total from 5-fold cross-validation repeated 20 times). Aggregate predictions were generated using a majority vote approach, while aggregate probabilities were calculated by averaging the predicted probabilities across all models.

On the independent test set, the CatBoost model achieved an accuracy of 0.63, an AU-ROC of 0.70, and an AU-PRC of 0.89. These results underscore the model’s ability to generalize to a distinct population, highlighting its potential utility in predicting CRC and adenoma outcomes based on GM data.

**Figure 3.**
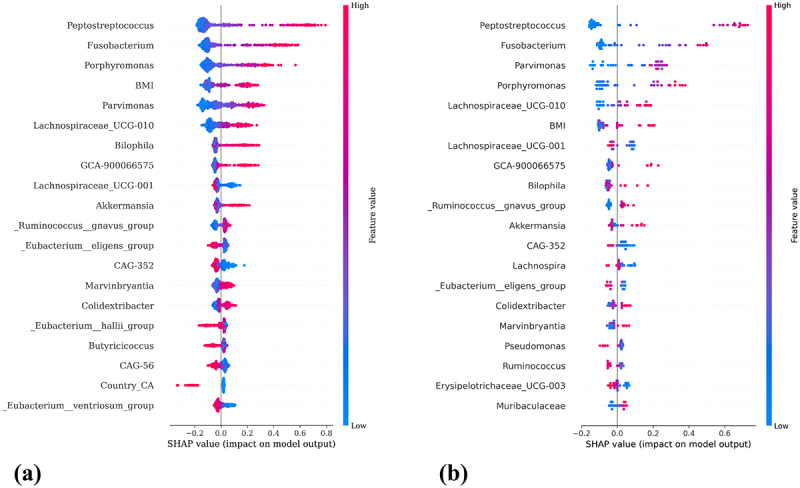
SHAP summary plots illustrating feature relevance for the classification of CRC and adenoma. (a) SHAP summary plot for the training dataset, showing the 20 most important features contributing to model predictions, which together account for 47.99% of the total cumulative SHAP importance. (b) SHAP summary plot for the independent test dataset, where the top 20 features account for 52.14% of the total cumulative SHAP importance. Each point represents a patient, with the horizontal axis indicating the SHAP value (impact on model output), and the color representing the feature value (red for high, blue for low).

In Figure 3, the SHAP summary plots are presented for the training dataset (a) and the independent test dataset (b) are depicted, illustrating global feature importance in each case. Each point in the SHAP summary plot corresponds to an individual patient, providing local explanations of the model’s predictions by depicting the contribution of each feature to the model’s output. For the training dataset, Shapley values were averaged across all iterations for each subject, accounting to the 20 repetitions during the training phase.

The summary plots provide insights into the relative importance of each feature, with color coding to indicate feature values: red represents high feature value, while blue denotes lower values. Across both the training and independent test datasets, certain features consistently emerged as influential for CRC classification. Specifically, genera such as *Peptostreptococcus*, *Fusobacterium*, *BMI*, and *Parvimonas* were identified as key contributors to CRC predictions. Conversely, features like*Eubacterium eligens* and *CAG-352* exhibited an opposing influence, being more strongly associated with adenoma classification.

**Figure 4.**
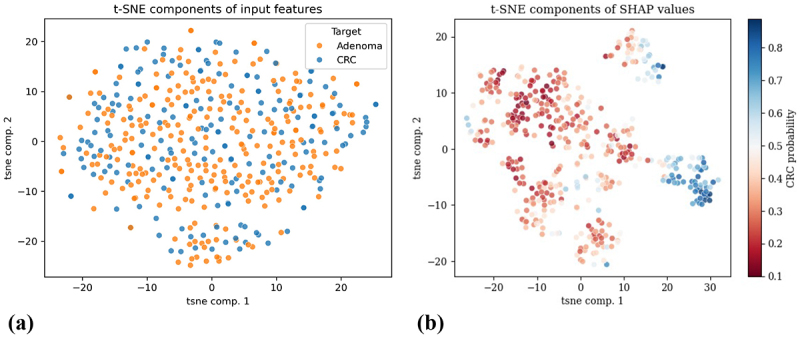
Dimensionality reduction with t-SNE on microbiome data. Subfigure (a) depicts the first two t-SNE components on microbiome data, while subfigure (b) represents the first two t-SNE components on SHAP values with color coding based on the probability of CRC.

When comparing feature relevance rankings for the training dataset to those of the independent test dataset, a notable consistency was observed. This alignment indicates that the key microbial taxa and clinical factors identified during training play also a significant role in model predictions on the independent dataset, underscoring the robustness and generalizability of these predictive features. The result of dimensionality reduction performed with t-SNE on GM data is displayed in Figure 4(a), showing the first two t-SNE components. Here, no distinct separation between the two group, adenoma and CRC, was observed. As a result, SHAP values were embedded and a t-SNE transformation was applied to these values. Figure 4(b) shows the resulting visualization, where subjects are plotted based on the first two t-SNE components derived from SHAP values, with colors representing their predicted probability of CRC. Notably, a clear separation among patients with high CRC probabilities and those with low probabilities was revealed, demonstrating the discriminative power of SHAP-derived embeddings.

**Figure 5.**
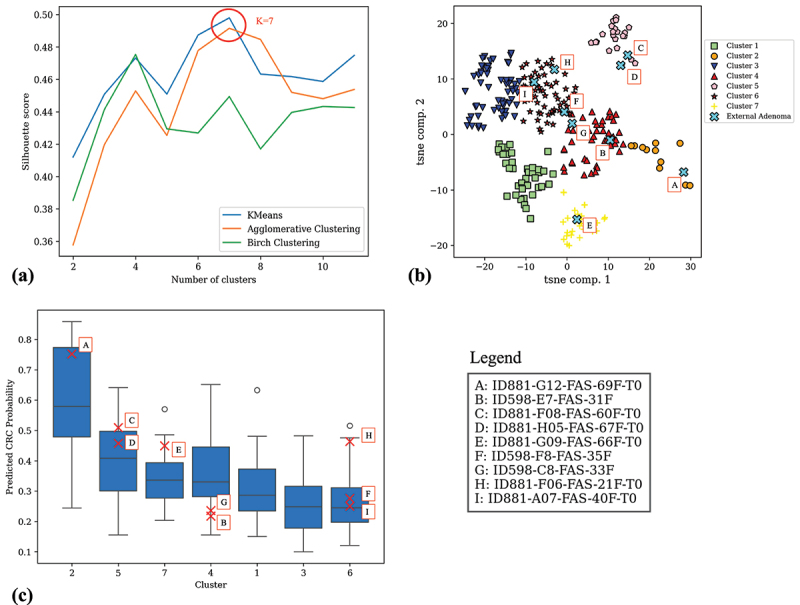
Clustering analysis of subjects with adenoma on SHAP values. (a) Silhouette score comparison of K-means, Agglomerative clustering, and Birch clustering. (b) t-SNE visualization of adenoma patients in the SHAP embedding, with cluster assignments for the training dataset and external adenoma patients projected into the SHAP space using a KNN model. (c) Box plot showing the distribution of predicted CRC probabilities across the different clusters, with red X’s marking the probabilities of adenoma patients from the external test set.

Given the clear separation observed among patients, the clustering analysis was focused exclusively on adenoma patients using the first two t-SNE components derived from the SHAP values. Three clustering methods were applied: K-means, Agglomerative Clustering, and Birch Clustering. As shown in Figure 5(a), K-means provided the optimal clustering results, achieving the highest silhouette score of 0.50, indicating well-defined cluster separation. The optimal number of clusters for K-means was determined to be seven.

In Figure 5(b), the adenoma patients in the SHAP embedding are visualized, clustered according to the K-means analysis. Additionally, the distribution of CRC probabilities predicted by the model across the different clusters is presented in Figure 5(c). Notably, Cluster 2 contains a distinct subgroup of adenoma patients exhibiting a significantly higher probability of CRC, followed by Clusters 5, 7, and 4, which showed progressively lower probabilities.

Furthermore, in Figure 5(b), adenoma patients from the independent test dataset were projected into the SHAP embedding derived from the training dataset using a k-nearest neighbors (KNN) model. The KNN model was fitted on the SHAP values from the training dataset to learn the mapping to t-SNE space. Subsequently, t-SNE coordinates for the external adenoma subjects were predicted.

In Figure 5(c), the probabilities for the adenoma patients from the external dataset are marked with red X’s, highlighting their positioning within the distribution of CRC probabilities across clusters.

**Figure 6.**
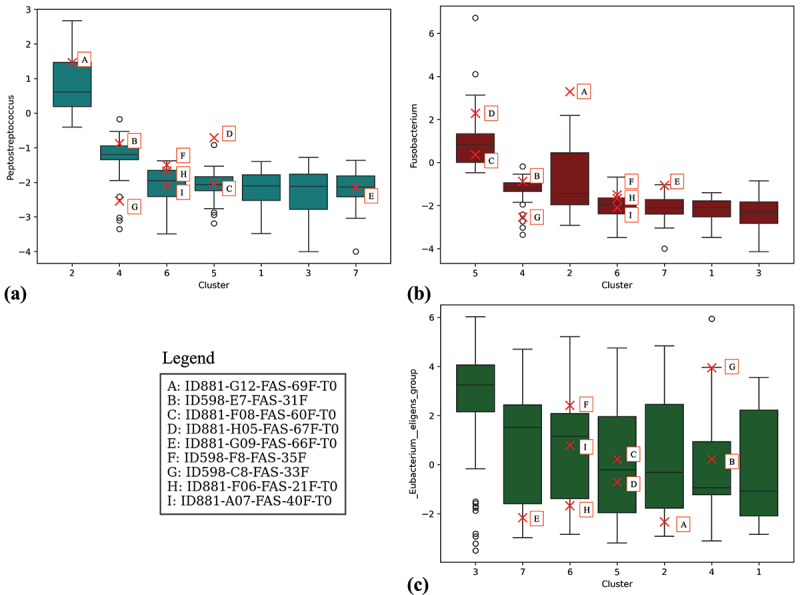
Boxplots showing the distribution of the relative abundance of *Peptostreptococcus*, *Fusobacterium* and *Eubacterium_eligens_group* bacteria across clusters. Red X’s represent the values for adenoma patients from the independent test set.

In Figure 6, the distributions of the two most relevant bacterial genera for the model, according to Figure 3—*Peptostreptococcus* and *Fusobacterium*, were plotted, alongside the *Eubacterium eligens group*, which exhibits an opposite trend. The SHAP summary plots indicate that as the abundances of *Peptostreptococcus* spp. and *Fusobacterium* spp. increase, the model’s prediction lean toward CRC, while higher abundances *Eubacterium eligens group* were associated with a decreased CRC probability. The red X’s in each plot represent the values of adenoma patients from the independent test set for these features. Notably, Cluster 2, which was previously identified as having the highest predicted probability of CRC, also shows the highest abundance of *Peptostreptococcus* spp. In detail, the adenoma patients in this cluster exhibits *Peptostreptococcus* spp. and *Fusobacterium* spp. levels above the median of the training distribution, while the same patient’s *Eubacterium eligens group* abundance is below the median of the training set.

### 3.1. Feature interaction analysis

SHAP interaction values were analyzed to explore pairwise relationships between features and their contributions to the adenoma-CRC classification model. The interaction values were calculated during cross-validation using the best-performing ML model and visualized to identify the most significant feature interactions.

In the supplementary material (Supplementary Figure S1), panel (a) presents the violin plot of SHAP interaction values, illustrating their distribution. To retain only the most robust interactions, a threshold based on the 95th percentile was applied, and features with fewer than 10 significant interactions were excluded from further analysis. Panel (b) of Supplementary Figure S1 shows the number of features retained as a function of the minimum number of interactions for three thresholds: 90th, 95th, and 99th percentiles. Using the selected threshold (95th percentile and at least 10 interactions), 40 features were identified, as represented in Supplementary Figure S1(c). This figure displays a bar plot where the x-axis represents the features and the y-axis their respective number of interactions. Finally, Supplementary Figure S1(d) depicts a histogram of the interaction counts for these retained features, highlighting the robustness of the selected interactions.

**Figure 7.**
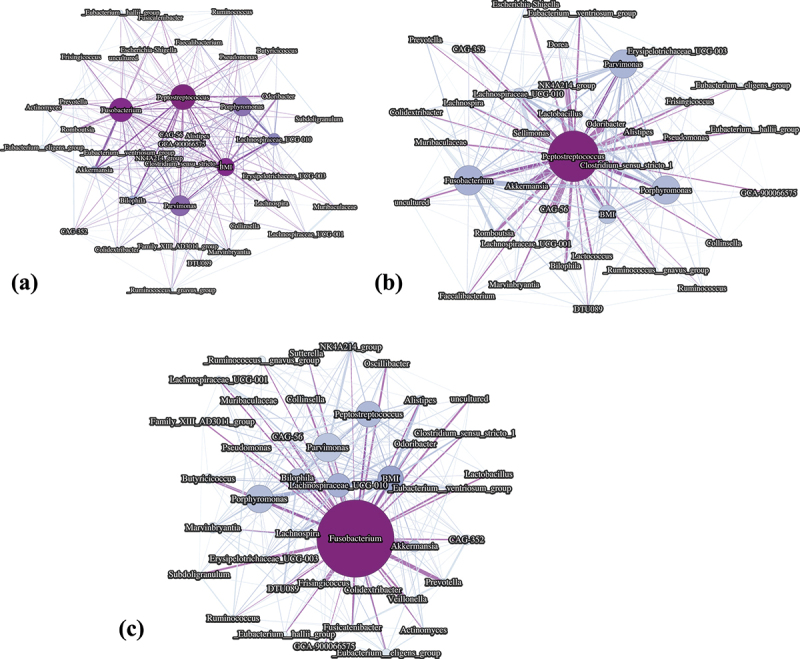
Weighted SHAP interaction networks. (a) Interaction network derived from all subjects in the training dataset, showing feature nodes sized by their number of interactions, colored by degree, and edges scaled by interaction intensity. (b) Network for subjects in Cluster 2 (high-risk group). (c) Network for subjects in Cluster 5 (second highest-risk group).

From this analysis, a weighted interaction network was constructed, with nodes representing the 40 retained features and edges representing SHAP interaction values. The network visualization was performed using the open-source software Gephi,^57^ employing the ForceAtlas2 layout. In Figure 7(a), nodes are sized proportional to their number of interactions, colored by their degree, and edges are scaled by interaction intensity. The analysis reveals that *Peptostreptococcus*, *Fusobacterium*, and BMI were the most central nodes, exhibiting strong interactions with other features in the network.

Subsequently, similar analyses were repeated for subsets of subjects from Cluster 2 (high-risk group) and Cluster 5 (second highest-risk group). These clusters were defined based on t-SNE embeddings derived from SHAP values and clustering analyses, as detailed earlier. For Cluster 2, 39 features with at least 10 significant interactions above the 95th percentile were retained, while for Cluster 5, 41 features met the same criteria. Supplementary Figures S2 and S3 (for Clusters 2 and 5, respectively) provide intermediate visualizations, including violin plots, feature selection thresholds, bar plots, and histograms.

The resulting networks are shown in Figure 7(b,c) for Clusters 2 and 5, respectively. In Cluster 2, *Peptostreptococcus* dominated the interactions, particularly with *Fusobacterium*, *Parvimonas*, and *Porphyromonas*, confirming its central role in this high-risk group. In Cluster 5, the hub feature was *Fusobacterium*, with interactions involving BMI, *Parvimonas*, *Porphyromonas*, *Peptostreptococcus*, and *Lachnospiraceae UCG-010*. These findings align with the trends observed in the boxplots in Figures 6(a,b).

## 4. Discussion

This study developed a XAI workflow to analyze GM 16S rRNA sequencing data data from 453 patients, with the aim of classifying patients with CRC from those with adenoma. The primary goal was to investigate the association between GM and the CRC onset, starting from adenoma development, a critical step toward understanding how personalized interventions could be designed within the context of precision medicine. To explore the relationship between GM dysbiosis and CRC, three ML learning classification models, namely XGBoost, Random Forest (RF) and CatBoost, were applied. The Random Forest (RF) algorithm operates as an ensemble of decision trees generated by repeatedly bootstrapping the training dataset.^45^ This method, combined with feature randomization during training, reduces correlation among the trees. Each decision tree makes independent predictions for individual observation, and their collective outputs are aggregated via averaging (for regression) or majority voting (for classification). In general, RF is known for offering easy tunability, minimal parameter requirements, resistance to overfitting, feature importance assessment during training, and unbiased estimation of generalization error. XGBoost, on the other hand, employs a collection of decision trees trained through iterative gradient boosting.^44^ This process iteratively addresses critical points within decision trees by constructing subsequent trees. XGBoost also incorporates sparsity-aware split finding, which exploits data sparsity patterns and determines optimal directions for splits when features are missing. Instead, CatBoost, as its name suggests, excels in handling categorical data within a gradient boosting framework.^46^ Gradient boosting entails iterative construction of decision trees, each enhancing the previous tree’s outcomes. CatBoost surpasses other decision tree-based methods by directly consuming a mix of categorical and non-categorical explanatory variables without preprocessing. It employs a technique called ordered encoding for categorical feature encoding, which considers target statistics from all prior rows to calculate replacement values for categorical features. Notably, all three ML classification models revealed a strong correlation between GM dysbiosis and CRC development, underscoring the potential of these models to elucidate the relationship between GM and CRC. However, while the findings highlight an association between GM imbalances and CRC, they do not address the directionality of this relationship – whether dysbiosis contributes to CRC development or whether CRC itself alters the fecal microbiota. Further longitudinal and mechanistic studies are necessary to explore this bidirectional relationship and clarify causality. Furthermore, SHAP analysis identified key microbiome variables positively associated with CRC, including *Peptostreptococcus* spp., *Fusobacterium* spp., and *Porphyromonas* spp. In line with these findings, the presence of *Peptostreptococcus anaerobius* in the GM of CRC patients has been linked to poor prognosis.^58^ Liu and colleagues recently demonstrated that *P. anaerobius* administration significantly reduced the efficacy of anti-PD1 therapy in CRC mouse models by inducing intratumoral myeloid-derived suppressor cells and stimulating their immunosuppressive activities, thereby impairing effective T-cell responses.^59^ Mechanistically, *P. anaerobius* activates the integrin $\alpha_{2}\beta_{1}$–NF-κB signaling pathway in CRC cells, which drives secretion of the chemokine CXCL1 and recruits CXCR2${\;}^{+}$ MDSCs (Myeloid-derived suppressor cells) into the tumor microenvironment. Additionally, *P. anaerobius* secretes a protein, LytC_22, that binds the Slamf4 receptor on MDSCs and upregulates *ARG1* and *iNOS* expression, further increasing their immunosuppressive activity. Moreover, both *in vitro* and *in vivo* studies revealed that *P. anaerobius* specifically colonizes CRC lesions and promotes resistance to oxaliplatin by promoting MDSC infiltration. In detail, the recruited MDSCs secrete elevated levels of IL-23, which triggers epithelial – mesenchymal transition in tumor cells via the Stat3 pathway, thereby contributing to chemoresistance.^58^ Similarly, *Porphyromonas* spp. have been found enriched in fecal and tissue samples from CRC patients compared with those from both adenoma patients and healthy subjects. Notably, *P. gingivalis* has been shown to increase tumor volume in the *ApcMin/+* mouse model, primarily through NLRP3 inflammasome activation, promoting CRC progression.^60^ Moreover, as widely reported in the current literature, *Fusobacterium nucleatum* resulted enriched in CRC patients, with higher intratumoral loads associated with recurrence, metastases and poorer prognosis.^61,62^ Mechanistically, *F. nucleatum* promotes CRC growth through its unique FadA adhesin, which binds to E” cadherin and activates Wnt/β-catenin signaling, triggering nuclear translocation of β-catenin and overexpression of oncogenes like c” Myc and Cyclin D1.^63^ Zepeda-Rivera *et al*. performed large-scale culturing, whole-genome sequencing, and comparative genomic analyses of *Fusobacterium nucleatum* strains from human CRC lesions and non-cancerous oral sites, uncovering CRC-enriched genetic features. They identified two distinct clades – C1, largely confined to the oral cavity, and C2, which predominates in the CRC tumor niche. Notably, only clade C2 drove tumor formation and shifted intestinal metabolism toward elevated oxidative stress in a CRC animal model. Comparative genomics revealed cumulative genetic adaptations that underlie C2’s pathoadaptation to the CRC microenvironment, establishing clade C2 as a highly virulent subgroup of *F. nucleatum* and a prime focus for mechanistic studies and CRC-targeted therapeutic development.^64^

Conversely, SHAP analysis identified a negative association between CRC and the *Eubacterium eligens* group as well as other members of the *Lachnospiraceae* family. Several *Eubacterium* species are known butyrate producers, a short-chain fatty acid essential for maintaining energy homeostasis, regulating colonic motility, modulating immune responses, and suppressing intestinal inflammation.^65^ Consistently, *E. eligens* has been found to be depleted in multiple CRC stages, supporting the rationale for exploring *Eubacterium* spp. as potential therapeutic agents for CRC. Notably, Feng *et al*. have secured patent rights for the use of *E. eligens* strains in the CRC treatment.^66^ Additionally, members of the *Lachnospiraceae* family, such as *Ruminococcus* spp., *Blautia* spp., and *Dorea* spp. were enriched in normal colon tissues compared to adjacent tumoral tissues in CRC patients. Finally, these bacteria have shown the capability to promote immune surveillance by enhancing the function of CD8+ T cells, acting as immune sentinels in colon tissue.^67,68^

In detail, this model demonstrated consistent predictive performance when tested on an independent dataset, underscoring its robust generalizability. This dataset, comprising Italian patients, exhibited microbiome profiles distinct from the training data, but the model maintained high predictive accuracy. Notably, the SHAP summary plots for the independent test set closely reflect those of the training data, indicating that the key features retained their importance in both datasets. This consistency highlights the robustness of these characteristics as markers of CRC risk, suggesting that they are reliable biomarkers in diverse populations. The consistent identification of bacterial genera significantly associated with the CRC risk suggests their potential utility as biomarkers for tracking the progression of adenoma to CRC. Such biomarkers could play a pivotal role in facilitating earlier detection and allowing more targeted, personalized interventions, advancing the precision medicine paradigm in clinical practice. Furthermore, by embedding SHAP values and applying the t-SNE transformation, a clear separation between patients with high and low probabilities of CRC was identified. Subsequent clustering analysis of adenoma patients revealed a subgroup with a significantly higher probability of developing CRC. This “higher-risk” subgroup, particularly those in Cluster 2, displayed elevated levels of *Peptostreptococcus* spp. and *Fusobacterium* spp., along with reduced levels of *Eubacterium eligens group* spp.,—a microbial profile strongly indicative of increased CRC risk. Although these findings are promising they remain preliminary and further case studies are needed to validate the reproducibility and robustness of these risk profiles.

To further explore the clinical relevance of the identified adenoma clusters, the distribution of key demographic variables, including age, BMI, gender, and country of origin were examined. While Cluster 1 exhibited slightly higher BMI values and Clusters 2, 3, and 7 showed a modest predominance of male subjects, no clear pattern emerged that could explain the stratification observed in predicted CRC risk (Figure 5c). Notably, these findings suggest that the SHAP-based clustering reflects underlying microbiome signatures rather than demographic heterogeneity. Supporting plots are provided in Supplementary Figures S4–S6.

Additionally, a SHAP-based network analysis was conducted to unravel the complex interactions between microbiome features and their influence on CRC risk. Weighted interaction networks, constructed from SHAP interaction values, revealed key microbial players and their interconnections, shedding light on the intricate dynamics of microbiome dysbiosis in CRC. Similar approaches have been successfully applied in other studies, such as Wang et al.,^69^ where SHAP interaction networks were used to elucidate interactions between multi-omics to predict plant complex traits.

The network analysis revealed that features such as *Peptostreptococcus* spp., *Fusobacterium* spp., and BMI served as central hubs with high degrees of connectivity. These nodes exhibited robust interactions with other features, suggesting their pivotal role in modulating CRC risk. Notably, *Peptostreptococcus* spp. was found to dominate the high-risk Cluster 2, with strong interactions involving *Fusobacterium*, *Parvimonas*, and *Porphyromonas*. These findings corroborate existing literature, which has consistently highlighted the oncogenic potential of these genera in CRC progression.

In contrast, Cluster 5, which represented the second-highest risk group, displayed a different interaction pattern. Here, *Fusobacterium* emerged as the primary hub, with moderate contributions from BMI and other genera such as *Parvimonas*, *Porphyromonas*, *Peptostreptococcus*, and *Lachnospiraceae UCG-010*. This divergence in interaction networks between clusters underscores the heterogeneity of microbiome contributions to CRC risk, suggesting distinct pathways of dysbiosis-driven carcinogenesis.

Recent studies have demonstrated that causal inference methodologies can be successfully applied to microbiome data to uncover potential causal relationships with complex diseases, including CRC.^70–73^ These approaches represent a promising avenue for future research aimed at complementing the explainability provided by SHAP-based models. While our SHAP interaction networks reveal important feature interdependencies and subgroup-specific risk profiles, integrating causal inference strategies could further elucidate the directional nature of host – microbe interactions and support the development of mechanism-driven interventions.

## 5. Conclusions

In conclusion, this study demonstrates the potential of XAI methodologies in uncovering the complex relationship between GM and CRC development. The application ML models, coupled with the interpretation of SHAP values, enabled the identification of key microbial biomarkers linked to the adenoma-CRC sequence. Notably, XAI analysis enabled the projection of adenoma patients into SHAP-defined spaces, revealing subgroups with an elevated risk of CRC, highlighting the value of this approach for targeted monitoring and early intervention. The results further emphasize the significant role of GM dysbiosis in CRC pathogenesis and the potential utility of these microbial signatures as biomarkers for CRC risk stratification.

The integration of SHAP-based interaction network analysis further underscores the utility of XAI in understanding complex feature interdependencies within the microbiome. By identifying central microbial hubs such as *Peptostreptococcus* and *Fusobacterium* and their interactions within high-risk subgroups, this approach provides a novel framework for exploring the underlying mechanisms of CRC progression. These findings highlight the potential of network-based insights to guide biomarker discovery and personalized intervention strategies, advancing the precision medicine paradigm in CRC risk management.

Although the external validation yielded promising performance, expanding the analysis to larger and multi-regional cohorts would strengthen the robustness and generalizability of the findings across diverse populations and clinical settings. Moreover, additional research, especially in microbial metabolome, is needed to establish the causal link between CRC and gut dysbiosis, to confirm the robustness of the proposed biomarkers in broader populations, and to evaluate their translational potential in clinical workflows. Additionally, the integration of complementary data types – such as metabolomics, transcriptomics, dietary information, and clinical phenotypes – represents a key avenue for enhancing the biological interpretability and mechanistic insight of microbiome-based predictions. Future prospective studies with multi-omics collection protocols could enable a more comprehensive understanding of host – microbe interactions and support more informative risk assessment frameworks. Lastly, while 16S rRNA sequencing offers a practical and scalable approach for taxonomic profiling, its limited resolution may preclude the detection of species-specific or functional signatures. Future work leveraging whole-metagenome shotgun sequencing could refine biomarker discovery and further enhance the clinical utility of microbiome-based models.

Finally, the subgroup-level insights revealed by XAI highlight the potential for developing personalized prevention strategies and enhancing CRC risk stratification in patients with adenomas. Prospective longitudinal cohorts that follow adenoma patients over time will be critical to confirm the validity of the SHAP-derived risk-stratification clusters.

## Supplementary Material

Supplemental Material

## Data Availability

The dataset used to construct the model presented in this study is available on Zenodo.^74^ The independent dataset used for model testing is available in the GEO repository and can be accessed online at https://www.ncbi.nlm.nih.gov/geo/query/acc.cgi?acc=GSE163366.

## Code availability

The code used to implement the methods and analyses presented in this study will be made available through a public GitHub repository upon publication.
