# Supplementary figures and images for: Personalized colorectal cancer risk assessment through explainable AI and Gut microbiome profiling
Source: Gut Microbes. 2025 Aug 4;17(1):2543124. doi: 10.1080/19490976.2025.2543124 (PMC12326576; doi:10.1080/19490976.2025.2543124)

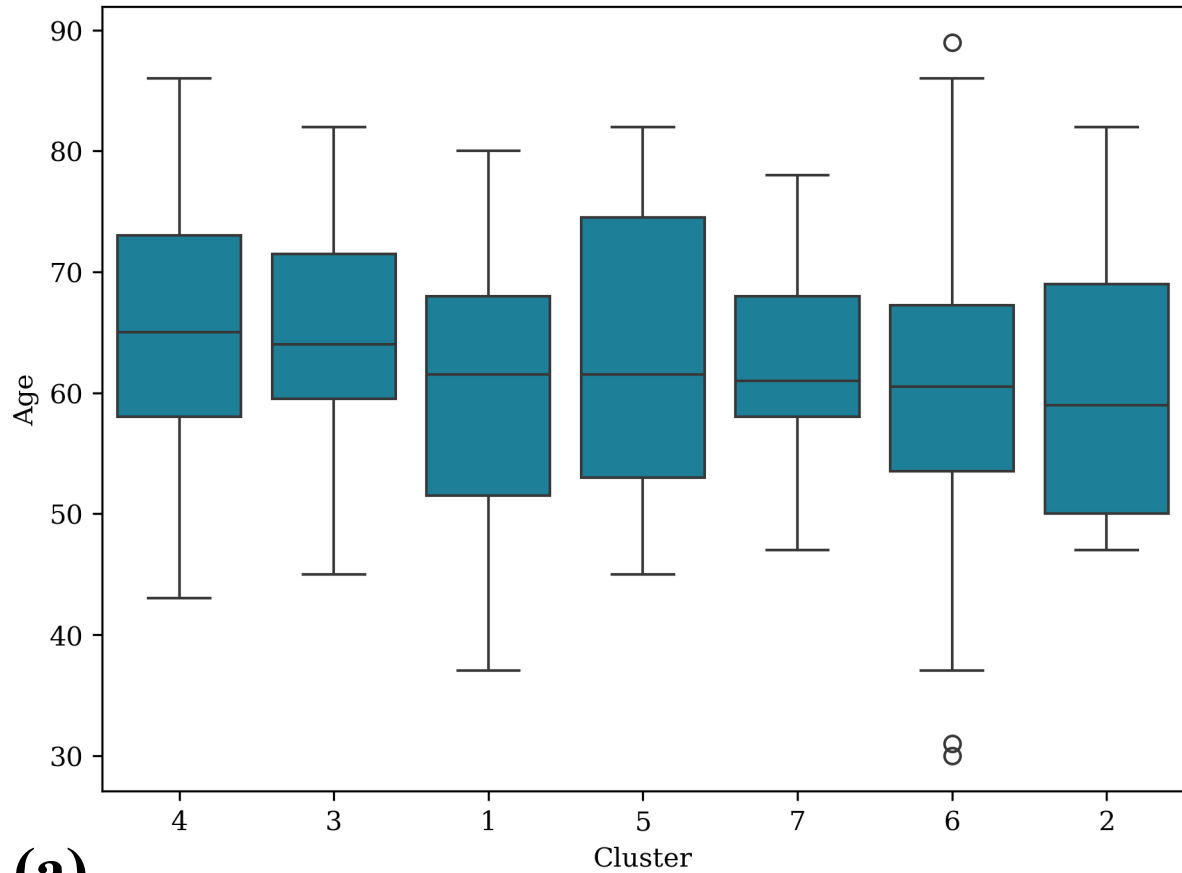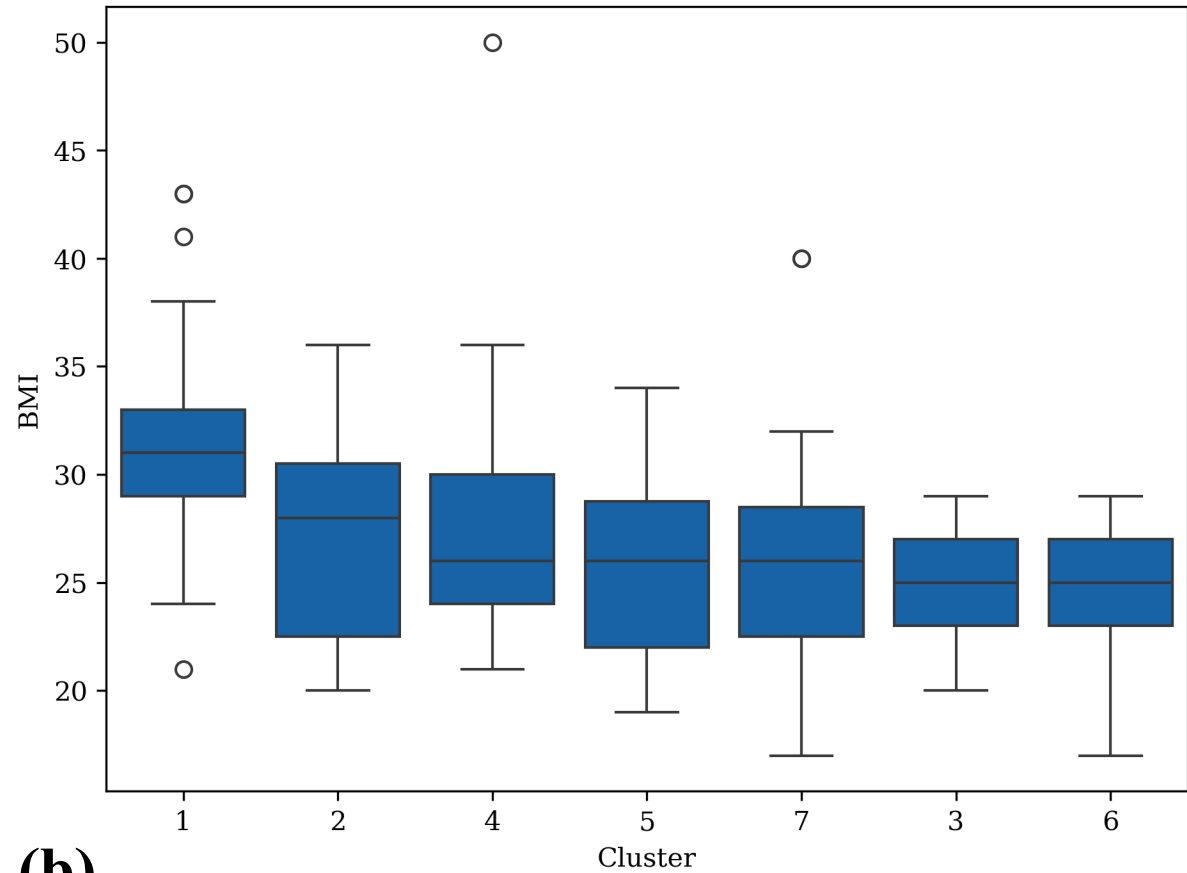

Supplement: Supplemental Material [file KGMI_A_2543124_SM2380.zip › images_supp/age_bmi.pdf]

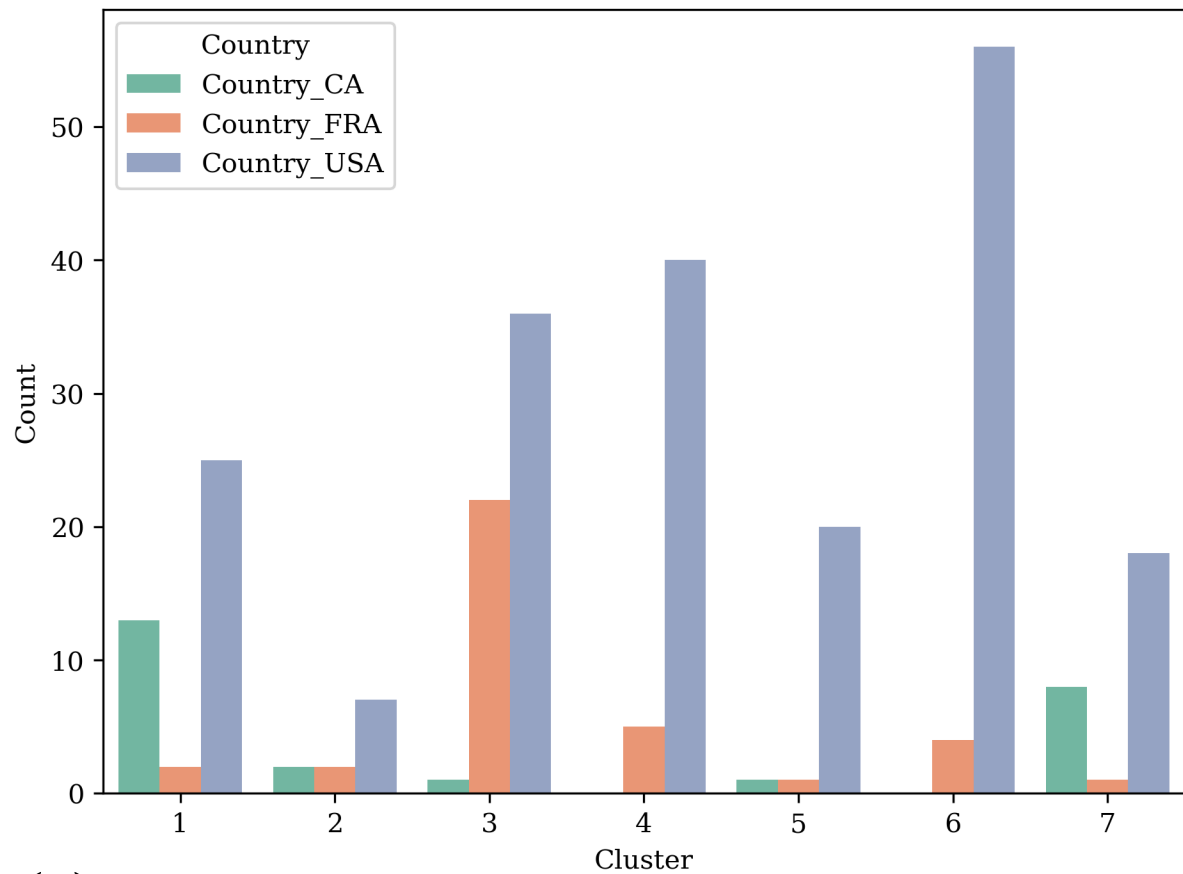

(a)

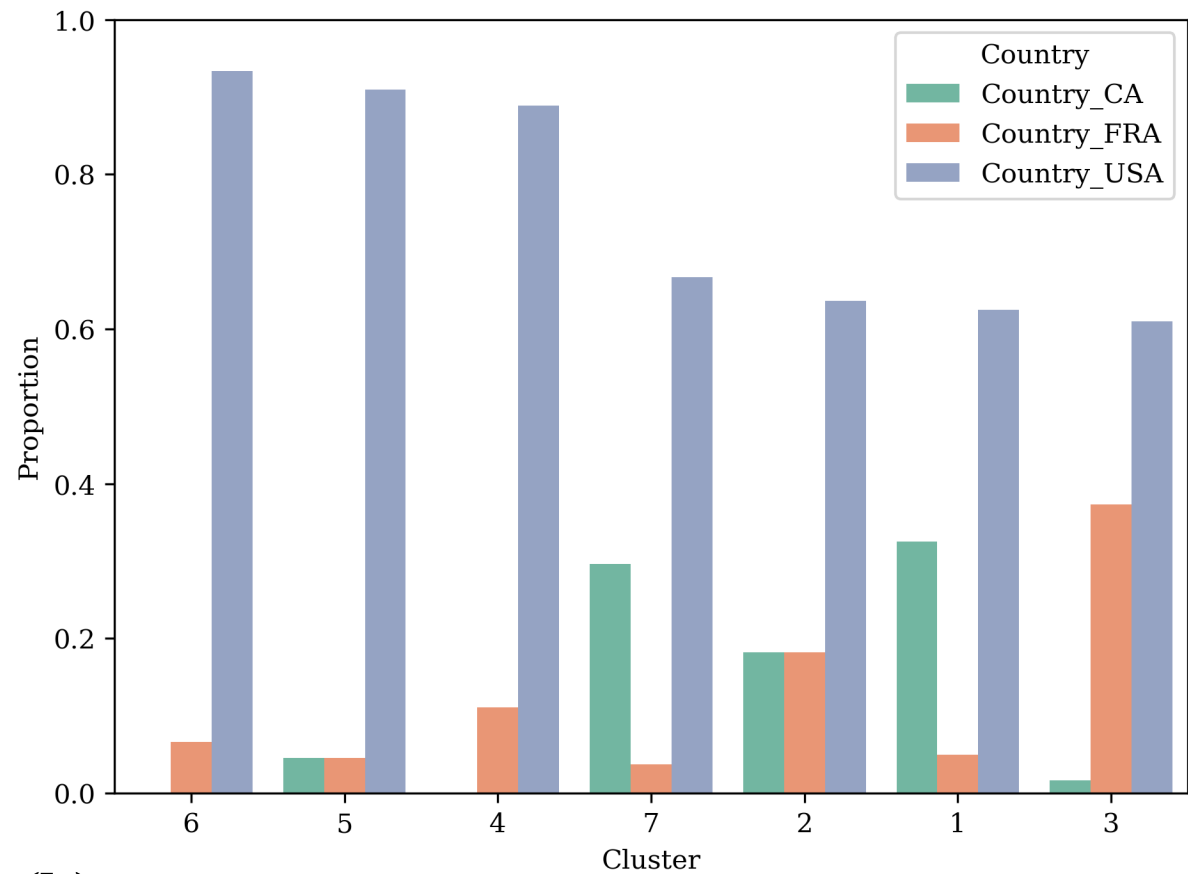

(b)

Supplement: Supplemental Material [file KGMI_A_2543124_SM2380.zip › images_supp/country.pdf]

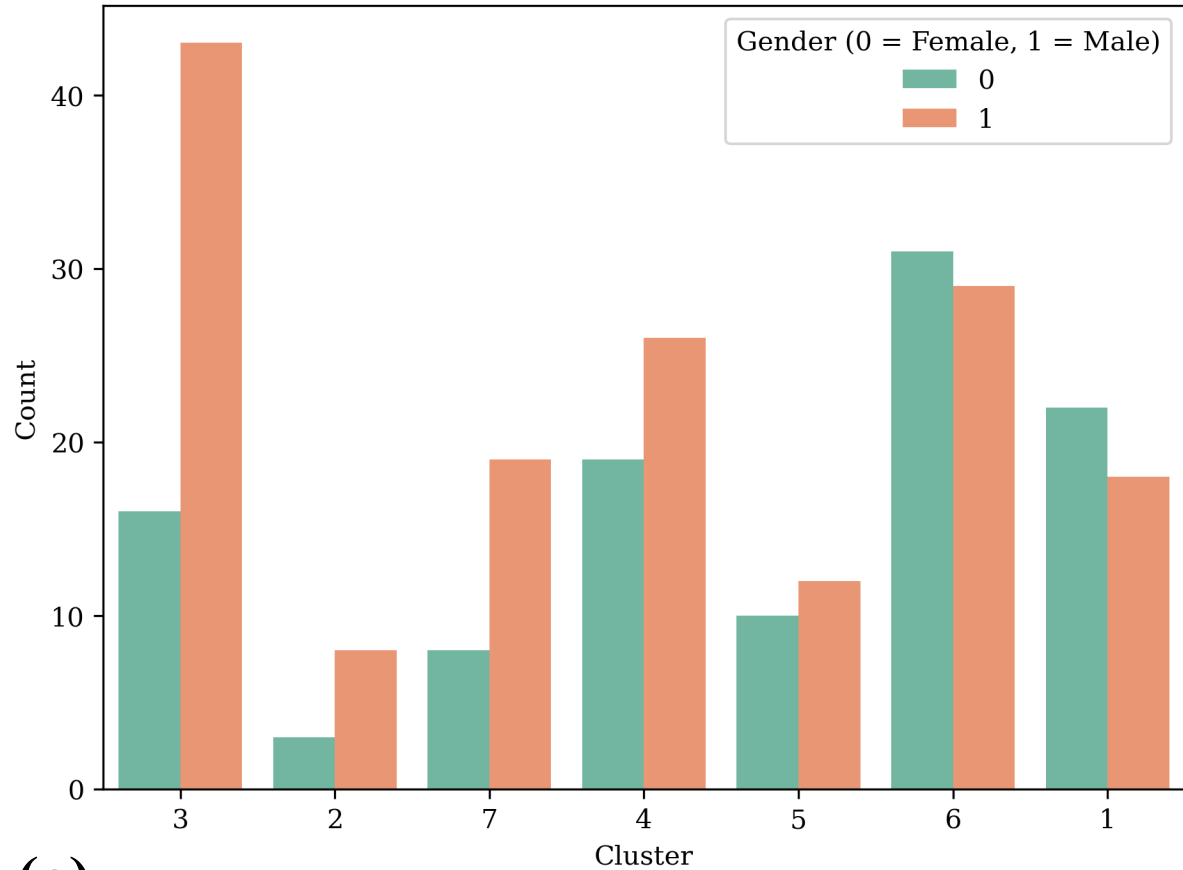

**(a)**

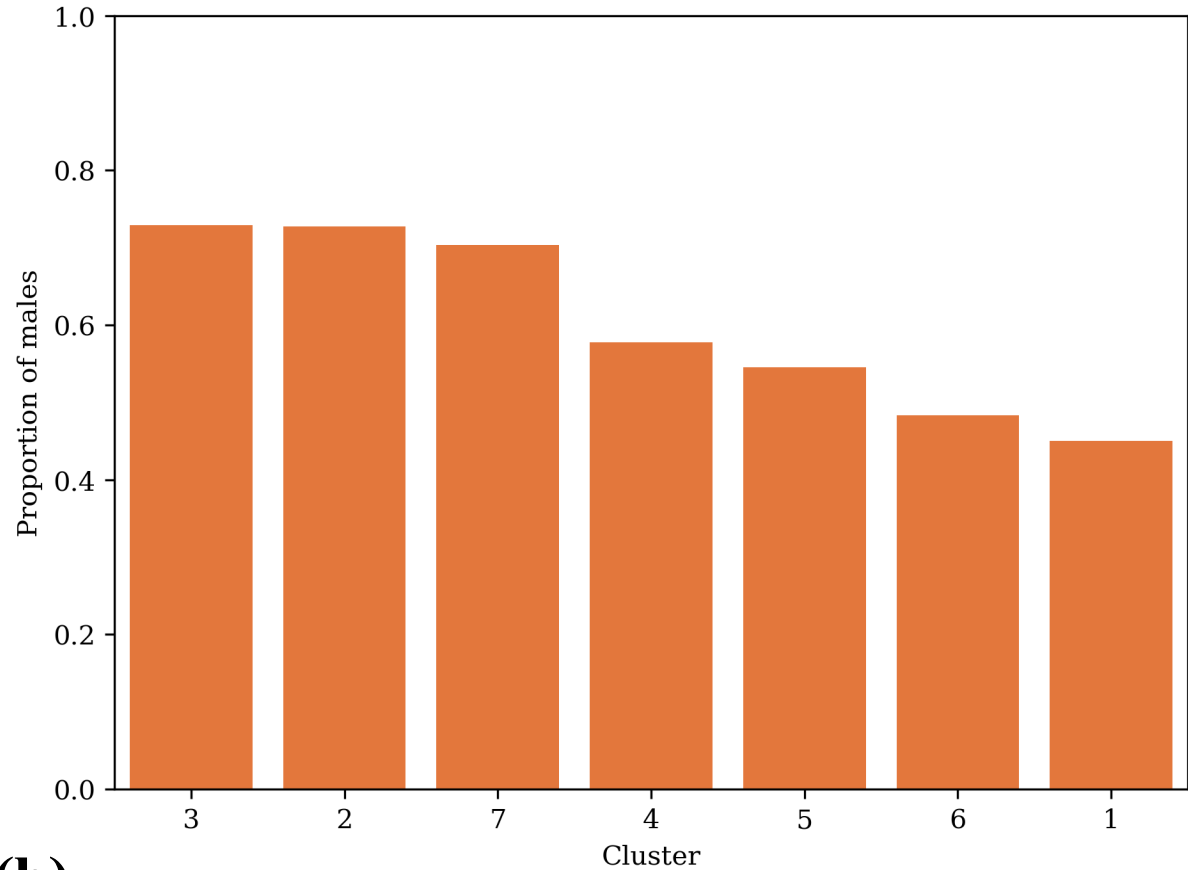

**(b)**

Supplement: Supplemental Material [file KGMI_A_2543124_SM2380.zip › images_supp/gender.pdf]

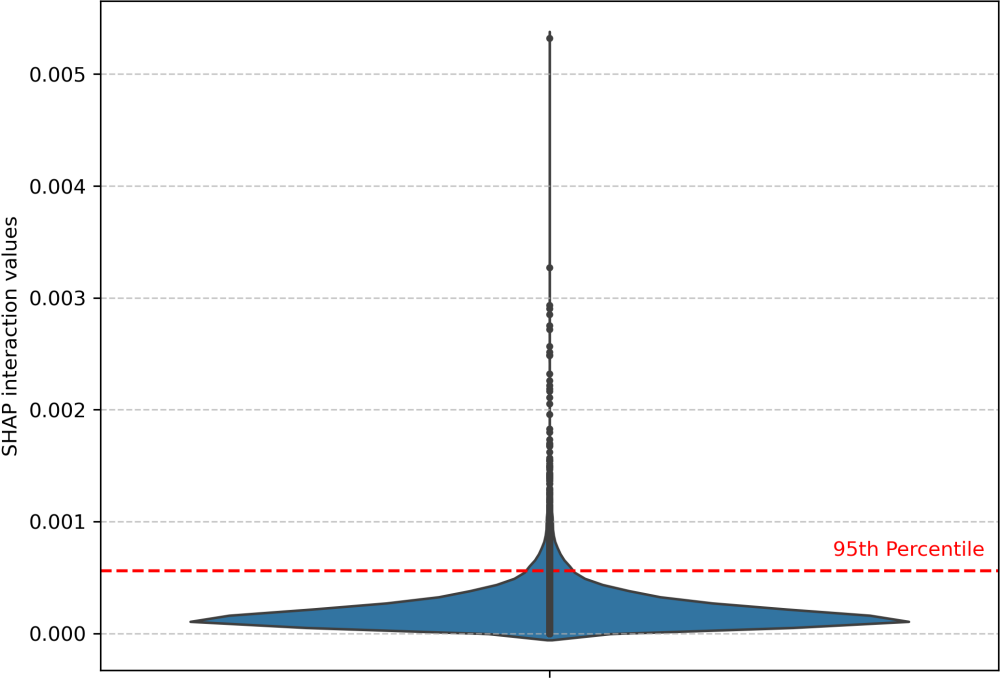

(a)

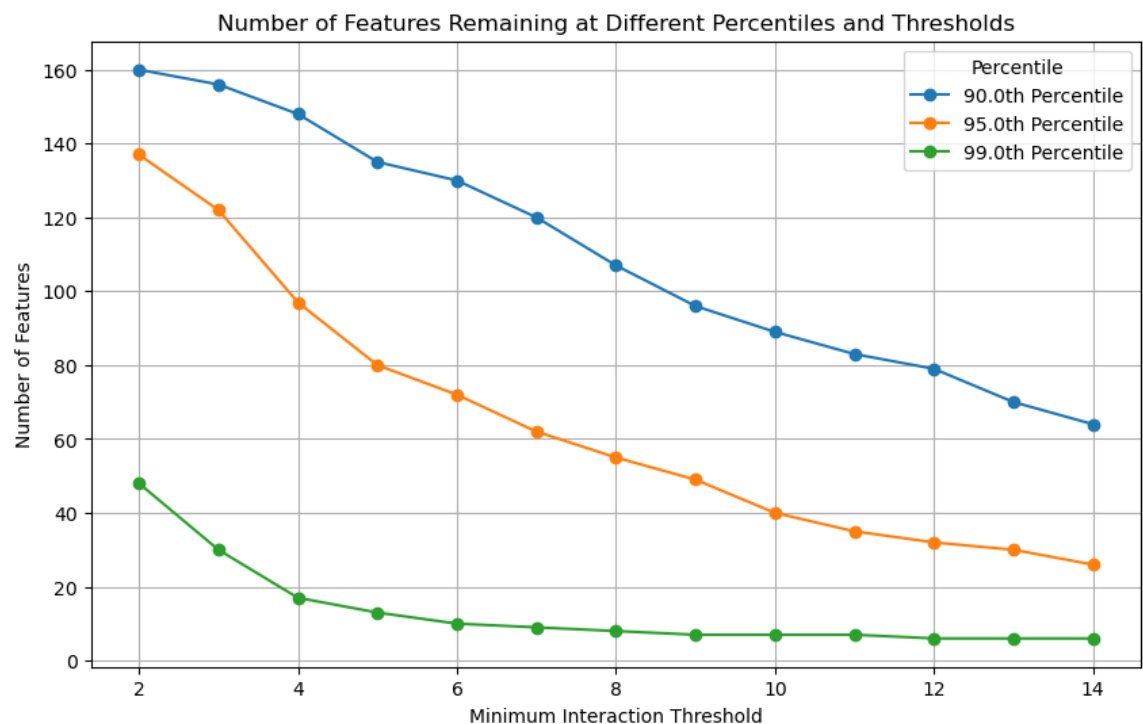

(b)

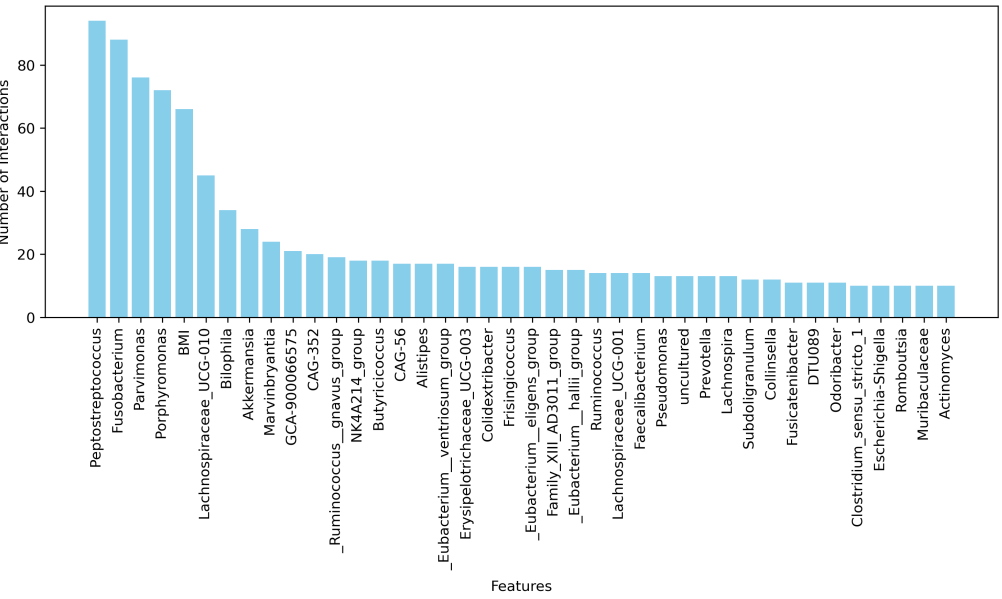

(c)

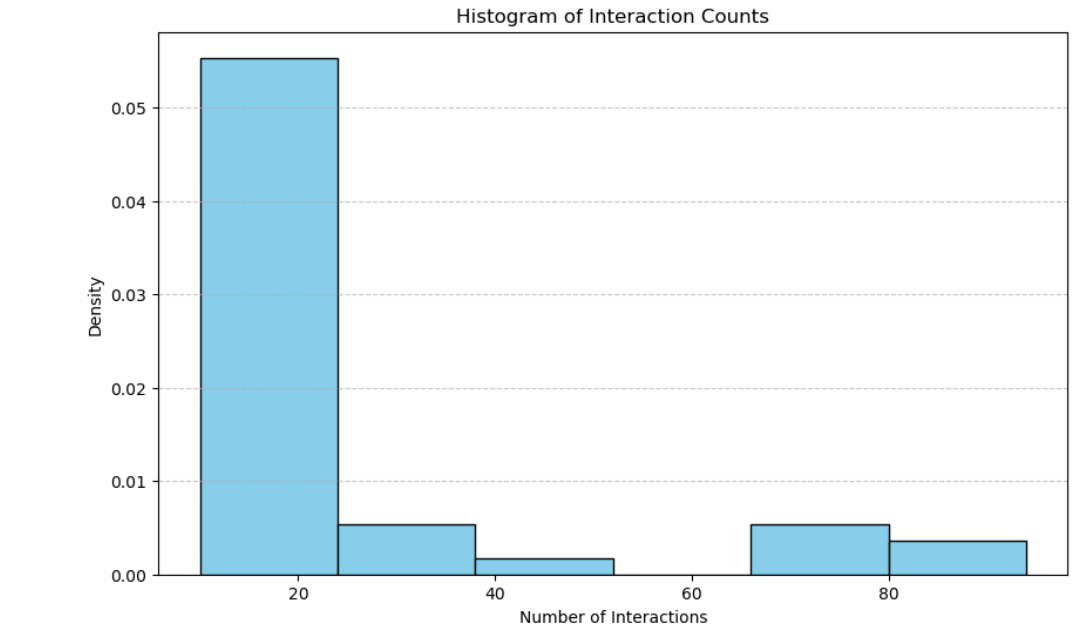

(d)

Supplement: Supplemental Material [file KGMI_A_2543124_SM2380.zip › images_supp/supp_adenoma.pdf]

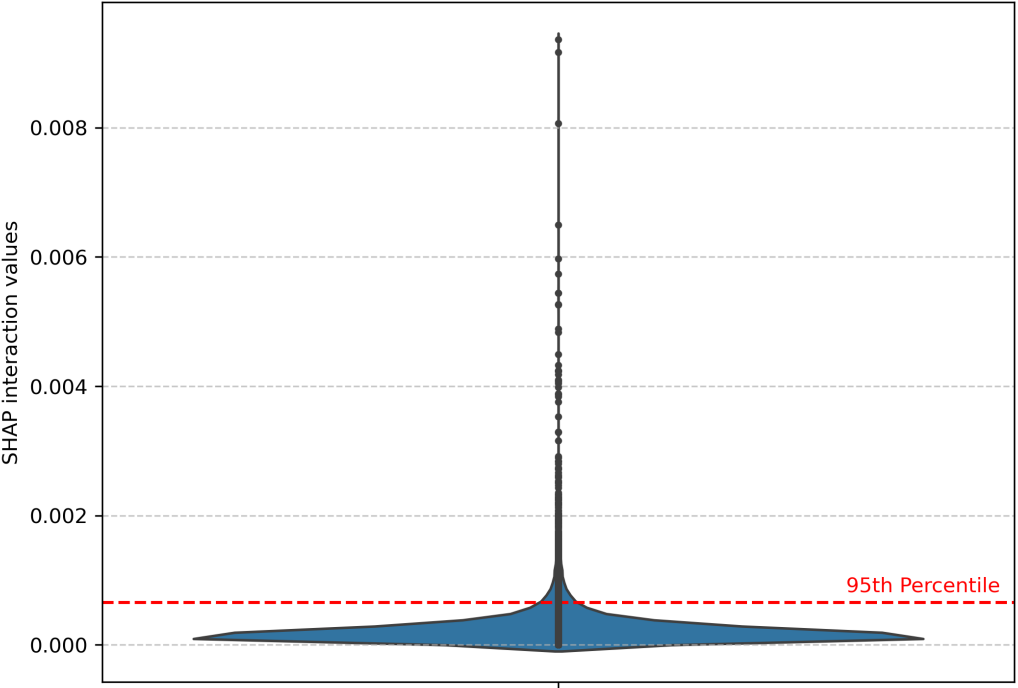

(a)

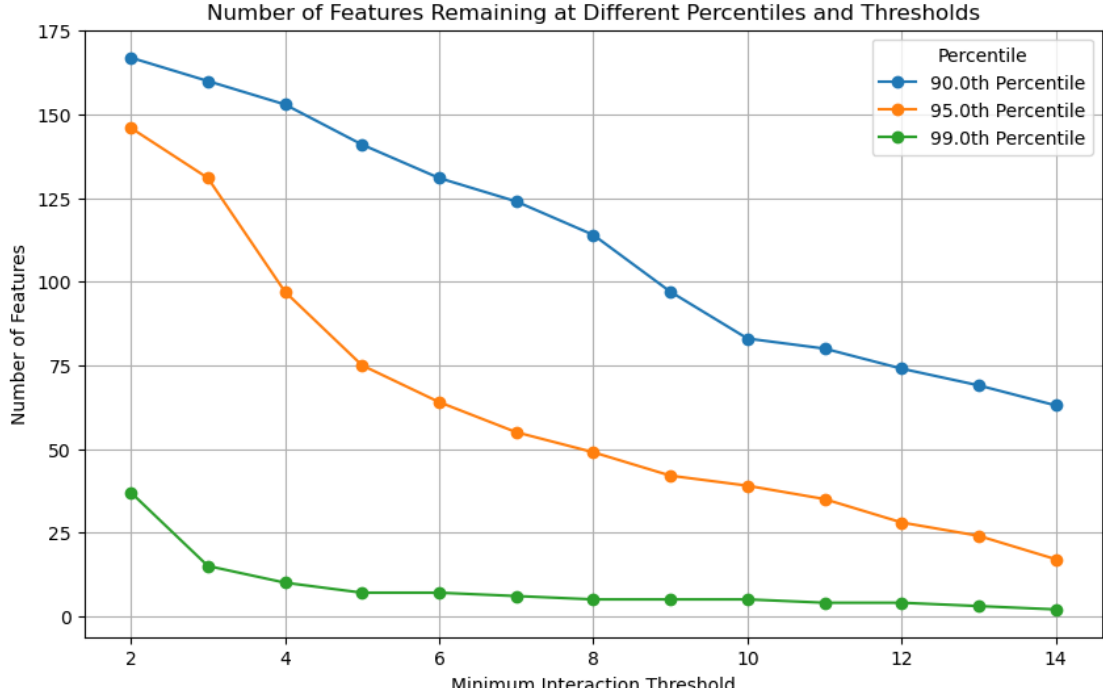

(b)

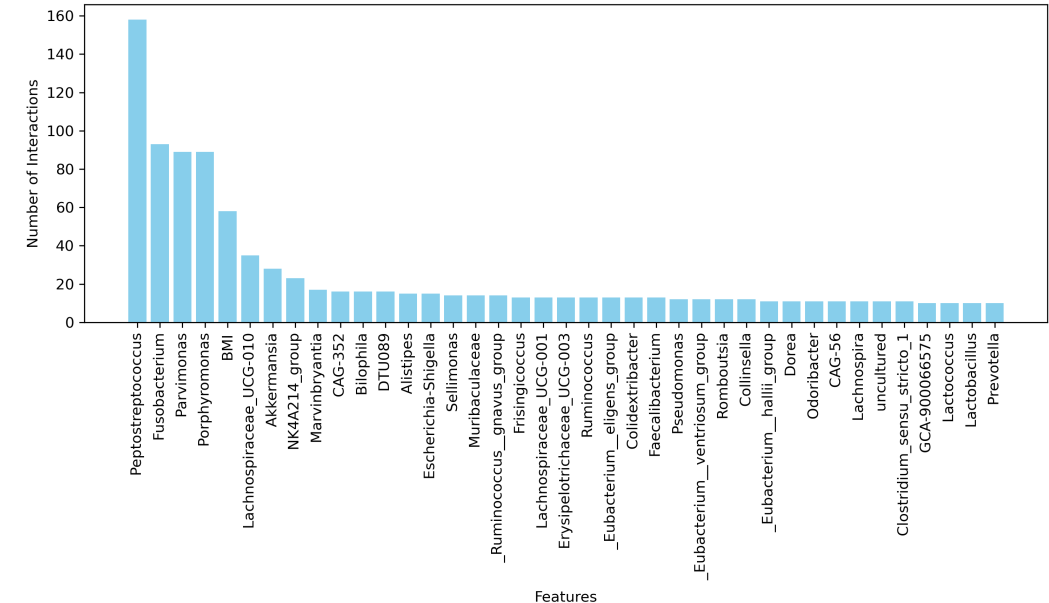

(c)

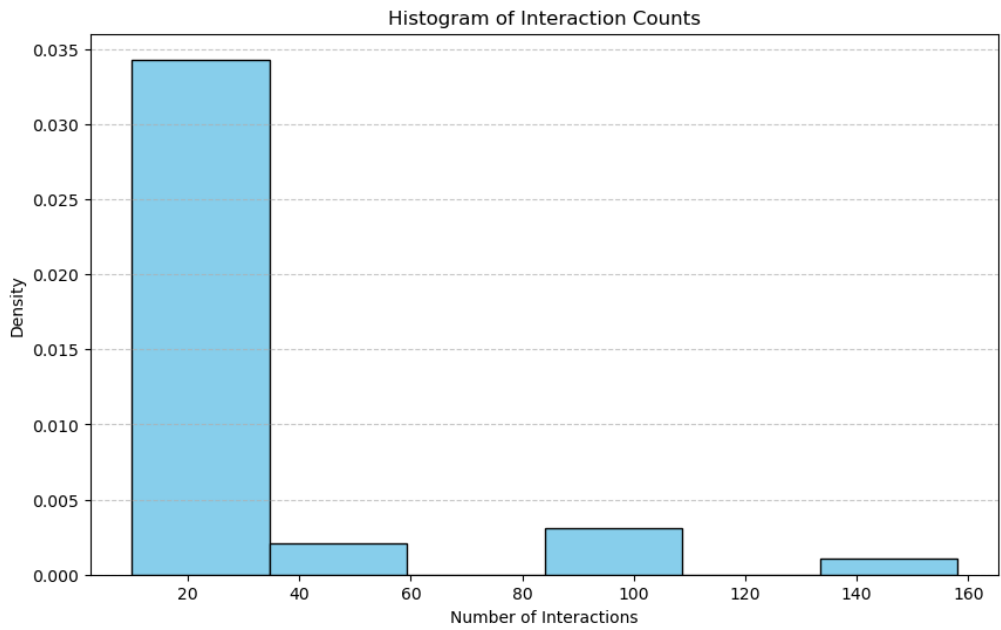

(d)

Supplement: Supplemental Material [file KGMI_A_2543124_SM2380.zip › images_supp/supp_adenoma_cluster2.pdf]

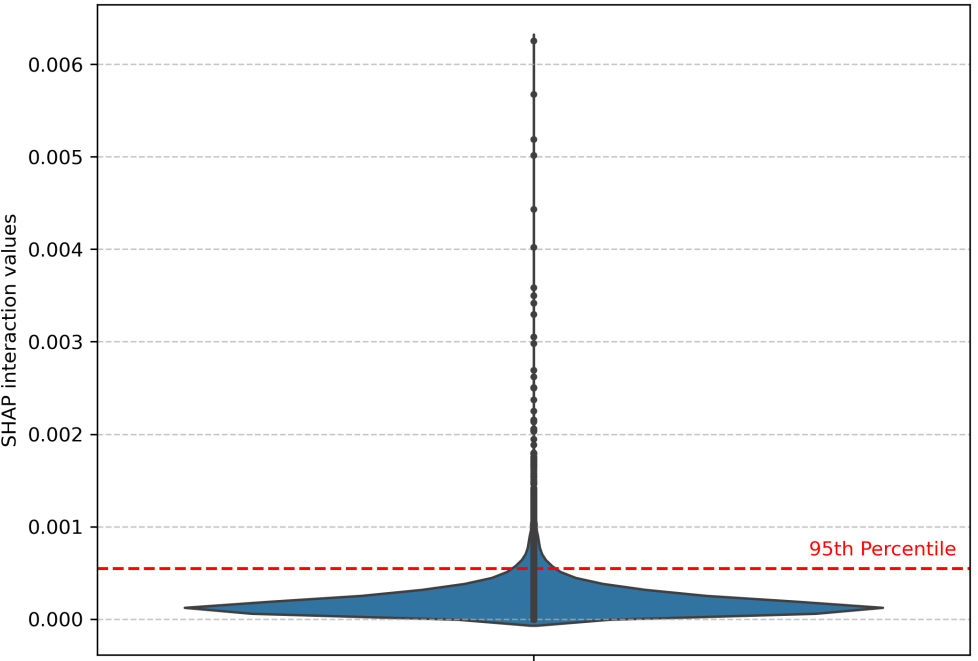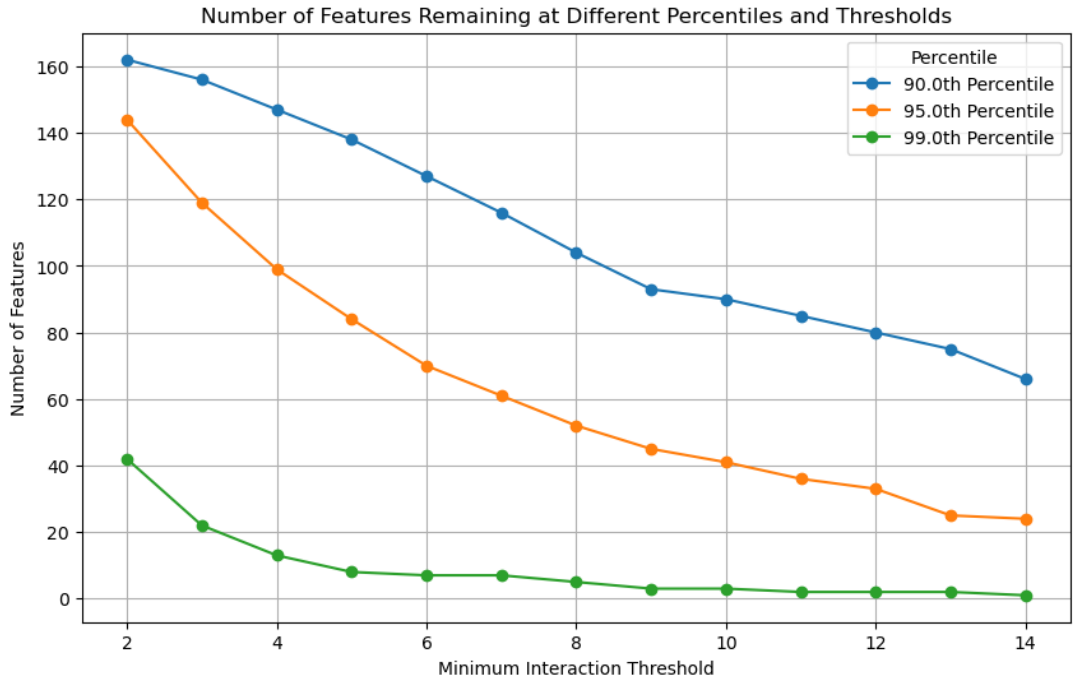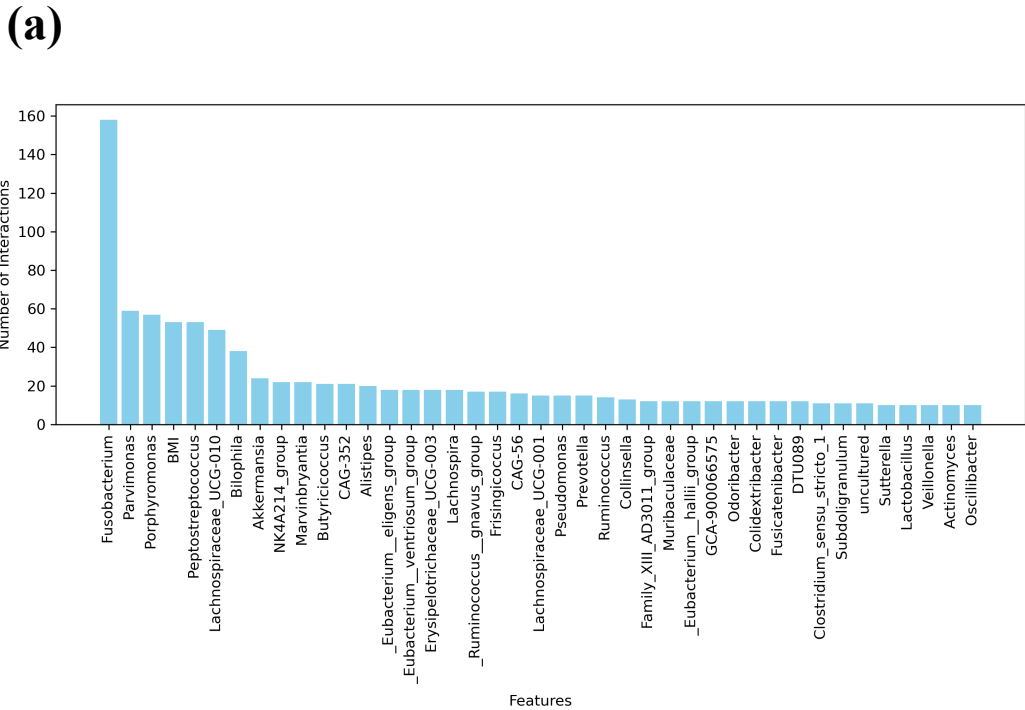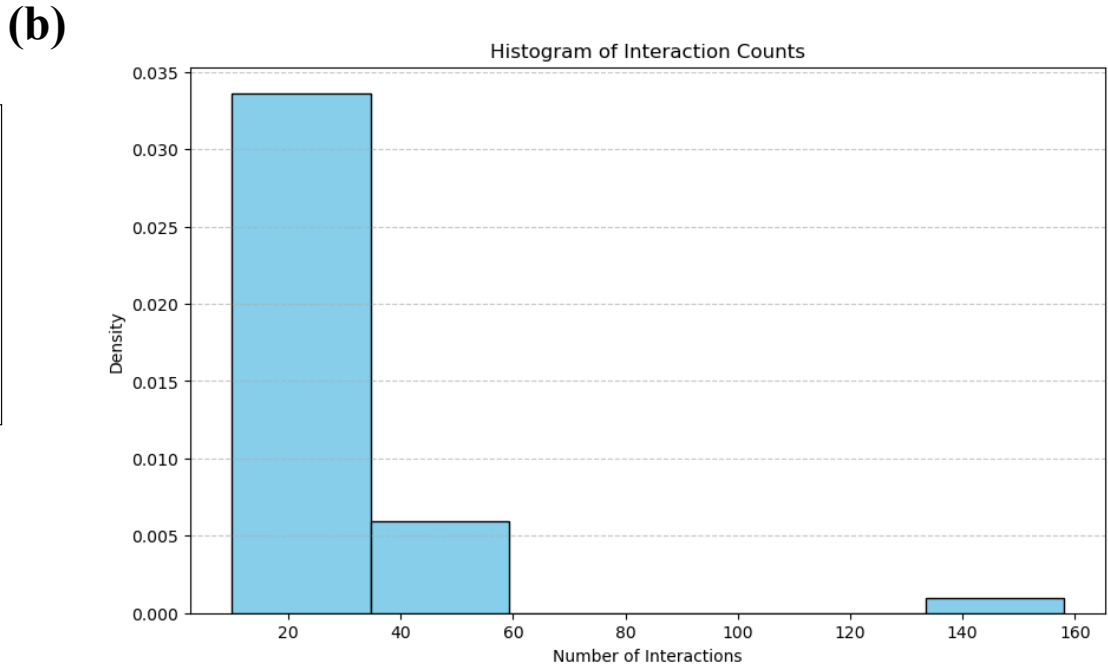

(c)

(d)

Supplement: Supplemental Material [file KGMI_A_2543124_SM2380.zip › images_supp/supp_adenoma_cluster5.pdf]
